# Impact of Gentle Touch Stimulation Combined with Advanced Sensory Stimulation in Patients in a Minimally Conscious State: A Quasi-Randomized Clinical Trial

**DOI:** 10.3390/life15020280

**Published:** 2025-02-11

**Authors:** Mirjam Bonanno, Antonio Gangemi, Rosa Angela Fabio, Marco Tramontano, Maria Grazia Maggio, Federica Impellizzeri, Alfredo Manuli, Daniele Tripoli, Angelo Quartarone, Rosaria De Luca, Rocco Salvatore Calabrò

**Affiliations:** 1 IRCCS Centro Neurolesi Bonino-Pulejo, Via Palermo, Cda Casazza, SS 113, 98124 Messina, Italy; mirjam.bonanno@irccsme.it (M.B.); antonio.gangemi@irccsme.it (A.G.); mariagrazia.maggio@irccsme.it (M.G.M.); federica.impellizzeri@irccsme.it (F.I.); angelo.quartarone@irccsme.it (A.Q.); roccos.calabro@irccsme.it (R.S.C.); 2 Department of Cognitive Science, University of Messina, 98122 Messina, Italy; rafabio@unime.it; 3 Department of Biomedical and Neuromotor Sciences (DIBINEM), Alma Mater University of Bologna, 40138 Bologna, Italy; marco.tramontano@unibo.it; 4 Unit of Occupational Medicine, IRCCS Azienda Ospedaliero-Universitaria di Bologna, 40126 Bologna, Italy; 5 A.O.U. Policlinico “G. Martino”, Via Consolare Valeria, 98124 Messina, Italy; manulialfredo@gmail.com; 6 A.O. Papardo, 98158 Messina, Italy; daniele.tripoli1994@gmail.com; 7 Department of Biomedical, Dental Sciences and Morphological and Functional Images, University of Messina, 98122 Messina, Italy

**Keywords:** neurorehabilitation, minimally conscious state, gentle touch, tactile stimulation, multisensory stimulation, Neurowave

## Abstract

Touch, particularly affective touch mediated by C-tactile fibers, plays a key role in emotional regulation and therapeutic interventions. However, tactile stimulation is underutilized in sensory stimulation (SS) protocols for brain injury patients, despite its potential to enhance consciousness and promote recovery through neural and autonomic regulation. Tools like the Neurowave enable advanced multisensory stimulation, including audio-visual and emotional inputs, but lack tactile components. Integrating gentle touch stimulation with such systems could further enhance neuroplasticity, improve heart rate regulation, and support recovery in patients with disorders of consciousness. In this study, twenty patients affected by minimally conscious state (MCS) were divided into two groups: an experimental group (EG n.10) and a control group (CG n.10). Both groups underwent standard neurorehabilitation, including conventional physiotherapy and speech therapy. The key difference was in the type of sensory stimulation. The EG received advanced sensory stimulation with the Neurowave system (which provides audio-visual and emotional sensory stimulation) in addition to gentle touch stimulation. The CG received conventional sensory stimulation without the Neurowave and neutral gentle touch stimulation. Each patient was evaluated by a multidisciplinary rehabilitation team, using clinical scales such as coma recovery scale—revised (CSR-R), level of cognitive functioning (LCF), before (T0) and after (T1) treatment. Additionally, heart rate (HR) and neurophysiological outcomes (P300) were also recorded for both groups (EG and CG). The MANOVA model revealed a significant interaction effect between group and phase on P300 latency (F (1, 18) = 10.23, *p* < 0.001, η^2^ = 0.09), indicating that the intervention involving gentle touch stimulation significantly influenced the P300 latency in the EG. The findings of this study contribute to our understanding of the therapeutic potential of emotional multisensory stimulation, which also includes gentle touch stimulation, in MCS rehabilitation. By demonstrating significant effects on both neurophysiological and functional measures, our results support the integration of tactile interventions into comprehensive neurorehabilitation programs.

## 1. Introduction

Touch is a fundamental sense that enables human connection and interaction. It can be broadly categorized into two types: proprioceptive and interoceptive, the latter also known as affective touch [1]. At the sensory level, affective touch is thought to be mediated by recently discovered unmyelinated C-tactile (CT) fibers, slow-conducting mechanosensitive nerves located in the skin [2]. These CT fibers respond optimally to slow, gentle stroking movements, such as light brushing, but are also sensitive to temperature changes and static touch [2,3]. In healthcare, touch is a critical component of many interventions, particularly in the field of rehabilitation, where it plays a key role in therapeutic techniques [4]. As a result, there has been growing interest in understanding the neurophysiological effects of touch [5,6,7]. However, relatively few studies have examined the role of touch-based stimulation, like gentle touch, as sensory stimulation for patients recovering from brain injuries [8]. In general, sensory stimulation (SS) refers to a range of techniques aimed at promoting arousal and behavioral responsiveness through the application of environmental stimuli, especially targeting patients with brain injuries [9]. While procedures may vary, they typically involve the presentation of simple, frequent, repetitive stimuli that often have autobiographical or emotional significance [9,10]. Currently, SS can be provided with innovative rehabilitation systems, like the Neurowave (Khymeia, Padova, Italy). This tool allows multisensory stimulation, including audio-video and emotional stimulation, as well as the registration of brain activity through the P300, by using an EEG cuff [10]. However, the Neurowave does not deliver any kind of tactile stimuli. This latter is often overlooked in the SS protocols, which are primarily focused on audio-visual stimuli. Notably, stimulation of CT fibers via gentle touch stimulation has been shown to influence emotional regulation and interoceptive awareness, primarily through the activation of the insula, a brain region involved in processing tactile information [5,6,7]. The insula’s involvement is likely responsible for the well-documented calming effects of gentle touch stimulation, which can induce parasympathetic responses such as heart rate reduction, mediated by oxytocinergic modulation [11].

Heart rate (HR), in particular, serves as an important marker of the body’s ability to adapt to changing conditions, including environmental factors, cognitive states, and autonomic regulation [12]. This is especially relevant in conditions such as Disorders of Consciousness (DoC), which often follow severe Acquired Brain Injuries (sABIs) [13]. DoCs include conditions like the Unresponsive Wakefulness State (UWS) and the Minimally Conscious State (MCS). Recovery from a DoC may result in an individual emerging from MCS (E-MCS) or, in some cases, achieving full recovery [14]. While the mechanisms underlying recovery from DoCs remain unclear, factors such as traumatic etiology, younger age, and lower injury severity have been associated with better outcomes [15]. According to Moattari and colleagues [16], gentle touch stimulation could increase patients’ levels of consciousness. The mechanistic rationale for this kind of therapy is that environmental stimulation (e.g., tactile stimuli) may enhance neural processing, support neuroplasticity, and thus promote reemergence of consciousness [8,9,16]. SS is postulated to reengage dormant subcortical networks that modulate arousal, resulting in the reactivation of cortical networks that mediate awareness. Because an increased level of consciousness is considered an indicator of disease improvement process in critically ill patients, particularly those with traumatic brain injury, any intervention or care that can increase the level of consciousness in such patients can improve the patient’s prognosis [8,9]. Evidence [8] indicated that providing regular SS decreases the risk of sensory deprivation by reducing the duration of Intensive Care Unit hospitalization and stress levels.

In the present study, we hypothesized that gentle touch stimulation in addition to advanced emotional SS with the Neurowave may induce significant modifications in neurophysiological (as per P300 latency) parameters and clinical outcomes in patients with MCS. Additionally, we hypothesize that the application of this kind of SS could have a positive impact on the HR of patients with MCS, in comparison to conventional SS. This study aims to explore the clinical, neurophysiological, and autonomic impacts of combining gentle touch stimulation with advanced sensory stimulation delivered through the Neurowave device in patients with MCS.

## 2. Materials and Methods

### 2.1. Population and Study Design

In total, 20 patients (5 females and 15 males with a mean age of 59.05) affected by MCS and their caregivers who attended the Semi-Intensive Care Unit of the IRCCS Centro Neurolesi “Bonino-Pulejo” (Messina, Italy), from May 2023 to September 2024, were enrolled in our study (see Table 1). All experiments were conducted according to the ethical policies and procedures approved by the IRCCS Centro Neurolesi Bonino-Pulejo Research Institute Ethics Committee (ID: IRCCSME-02-2023, 20 November 2023). All patients’ caregivers gave their written informed consent to participate in the study and data publication. Patients were randomly divided into two groups, with ten in each group. One group received experimental treatment (EG), while the other received conventional treatment, forming the control group (CG).

The allocation was based on the order of recruitment, following quasi-randomization criteria. This method involves predefined and reproducible rules, such as alternation (e.g., one participant assigned to EG, the next to CG) and temporal entry order. By avoiding discretionary decisions from researchers, quasi-randomization ensures a systematic yet impartial distribution of participants, reducing the risk of allocation bias while maintaining practical feasibility.

Patients were included in our sample if they had the following: (1) a diagnosis of MCS following an acquired brain injury (vascular or traumatic), according to clinical and neuroradiological findings; (2) an age range between 18 and 76 years; and (3) the presence of a caregiver. Patients were excluded if they had the following: (1) administration of sedatives; (2) presence of skin lesions on the thorax, upper and lower limbs, or abdomen; (3) active epilepsy.

### 2.2. Procedures

**Figure 1:**
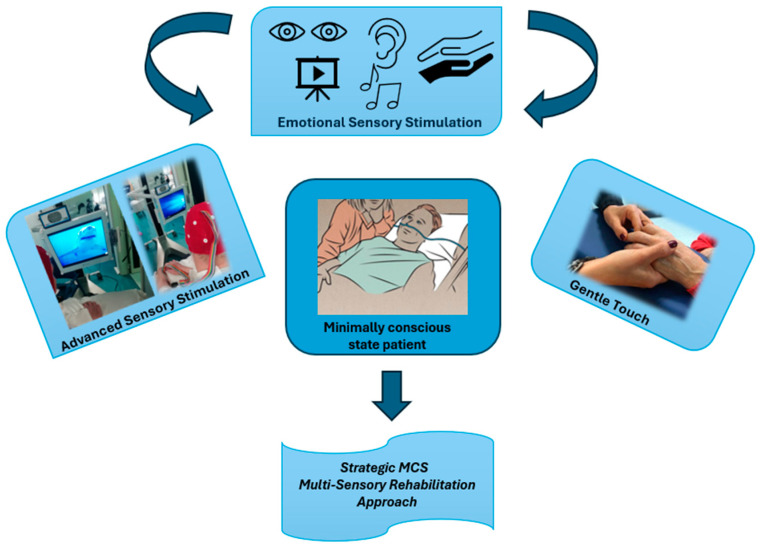
Schematic representation of audiovisual Neurowave stimulation plus gentle touch stimulation.

The enrolled patients received either advanced gentle touch stimulation with the Neurowave device (Neurowave, Khymeia s.r.l, Padua, Italy) in the experimental group (EG) or conventional sensory stimulation in the control group (CG). Both groups also received standard neurorehabilitation, which included conventional physiotherapy and speech therapy (see previous publications [10,17,18,19]). The EG received advanced sensory stimulation using the Neurowave (audio-visual stimulation) combined with gentle, emotionally enriched touch, while the CG received conventional sensory stimulation associated with neutral touch (see Figure 1).

Both groups followed a rehabilitation protocol consisting of one-hour sessions, three times a week, for 24 weeks (see Table 2 for details). The session duration was adjusted as needed based on patient fatigue and vital signs, monitored by nursing staff.

### 2.3. Outcome Measures

Clinical outcome measures were administered to all MCS patients involved in the study, before (T0) and after (T1) the rehabilitation protocols, by a multidisciplinary team (neurologist, psychologist, psychiatric therapist, physiotherapist, and speech therapist).

Specifically, patients were clinically evaluated using the coma recovery scale—revised (CSR-R) [20], which offers an in-depth assessment of consciousness and recovery by examining six specific functions: (i) auditory (e.g., responses to sound, such as following commands or localizing stimuli), (ii) visual (reactions to visual stimuli, including eye-tracking or fixation), (iii) motor (e.g., assessment involves observing voluntary motor actions such as purposeful movement, limb withdrawal in response to pain, or attempts to manipulate objects), (iv) oral-motor/verbal (e.g., verbal sounds, words, or purposeful mouth movements), (v) communication (e.g., considering ability to communicate through verbal or non-verbal means), and (vi) arousal, which is assessed based on the patient’s ability to remain awake or responsive to stimuli, including periods of eye-opening or alertness.

The level of cognitive functioning (LCF) was administered through observation and interaction with the patient. It is used to monitor and assess cognitive recovery by classifying the patient’s difficulties and residual abilities on an eight-point scale, where Level 1 indicates a complete absence of response to any stimulus; Level 2 indicates generalized responses (e.g., reflexes); Level 3 assesses specific reactions to stimuli, such as turning toward a sound or withdrawing from pain; Level 4 corresponds to inappropriate behavior, and difficulty with attention and focus; Level 5 indicates better response to commands but inappropriate actions and limited focus; in Level 6, responses are more appropriate; in Level 7, routine activities are performed automatically, with limited insight into their own condition; lastly Level 8 represents an independent patient with possible adaptations [21].

In addition, a nurse and neurophysiology technician, respectively, registered in both groups (EG and CG) heart rate (HR) and neurophysiological outcome (P300). The HR was evaluated before and after each conventional or not touch-based stimulation session by a nurse by using an electronic monitoring device (PM 60, Mindray Medical, Milan, Italy), to evaluate the level of relaxation for both groups. The neurophysiological evaluation was obtained by measuring the P300 signal with the Neurowave device, which was recorded in a resting state on the same day of the clinical assessment as well as at the end of the protocol.

### 2.4. Interventions

#### 2.4.1. Neurowave and ERPs Parameter Recording

Patients in the EG were treated and assessed with the Neurowave device [10,22,23]. Neurowave is a cutting-edge device that automates multi-sensory stimulation, such as images, videos, sounds, and personal memories, tailored to each patient’s needs. It is particularly suited for treating patients with DoC. For therapists, it is ergonomic, compact, and easy to move between beds. The Neurowave also allows simultaneous recording of various physiological signals, including the P300. P300 recordings were taken at the first and last EG sessions. ERPs were recorded using three Ag/AgCl electrodes placed along the scalp midline (Fz, Cz, Pz), following the International Ten-Twenty System, with additional electrodes for electro-oculograms placed near the eyes. Data were digitized at 256 Hz and filtered between 0.15 and 30 Hz, with a notch filter applied. Patients received intensive, repetitive, task-oriented sensory-motor stimulation, using personalized emotional audio-video content. Stimuli lasted 500 ms, with an 800 ms gap between each, and rare stimuli appeared 20% of the time in a random order. Each session lasted 60 min.

#### 2.4.2. Experimental Intervention: Advanced Sensory Stimulation Combined with Gentle Touch Stimulation

Before starting each multisensory training (advanced and not), the caregiver (which was included as a “co-therapist” in the multidisciplinary team) fulfilled the patients’ biographical format during a semi-structured interview focused on main aspects of the DoC patient’s life. The semi-structured interview was based on specific patients’ aspects of life, including (i) autobiographic experience, related to work activities and tasks, people who provided emotional support, key life events, and the most significant places in the patient’s life; (ii) personal identity that covered professional and domestic skills, lifestyle and sports activities, hobbies or interests, self-care routines, eating preferences or favorite dishes, and meaningful travel experiences; (iii) individual context, which is referred to favorite objects, preferred scents or fragrances, frequently used items, favorite colors, music and songs, familiar voices or recordings, photos or recordings of pets, and significant spaces or places; (iv) visual memories, including photographs or videos featuring familiar people, such as parents, children, and friends; and (v) relevant emotional events related to their life and their families. Altogether this information was used by the therapist to implement advanced multisensory training, enhancing the patient’s therapeutic experience.

The advanced multisensory training was carried out in a dedicated room by using the Neurowave device that allowed an automated administration of multi-sensory stimulation through personalized images, movies, sounds, and patient-specific memories.

In the EG, the gentle touch stimulation was performed manually in different body sites (i.e., wrists, hands, arms, and legs) by the physiotherapist previously trained to deliver tactile stimulation with desired force and velocity using hands. The level of the applied force (0.2 N) was chosen from the literature where Loken et al. [24] reported that CT fibers stimulated with a brush showed maximal sensitivity for movements characterized by a normal force on the skin of 0.2–0.4 N. Although force was not measured directly during each session, the physiotherapist, who performed gentle touch stimulation, was previously trained to maintain consistent touch by monitoring pressure quality through subjective feedback (e.g., attention to pressure and speed applied). The physiotherapist applied with their hands a bilateral constant static light skin-to-skin pressure in the proximity of different body parts, like wrists, hands, and lower and upper limbs.

#### 2.4.3. Conventional Multisensory Stimulation

MCS patients in the CG received conventional multisensory stimulation that is based on the premise that engaging different sensory modalities (sight, sound, and touch) can help to re-engage the brain’s neural networks. SS is provided to target specific regions of the brain responsible for processing each type of sensory input. The stimulation is structured, repetitive, and designed to provoke responses, including changes in HR, which might signal increased awareness.

The conventional SS training sessions were conducted in a dedicated room where the therapist used specific materials and information. During the sessions, the psychiatric technician and psychologist used a variety of audio-visual and tactile stimuli, both neutral and personalized. Audio-visual stimuli included colorful images, music videos, and material objects. Tactile stimulation involved activities such as touching and feeling objects with different textures (e.g., smooth, rough, soft, bumpy), handling textured items (e.g., numbers, letters, shapes), using sensory bins filled with materials like rice, sand, or beans, and manipulating materials of varying consistencies (e.g., glass surfaces, sea stones, dough, slime). These activities also incorporated temperature variations, such as using warm and cold water, to enhance sensory engagement. By incorporating specific techniques and tactile-centric activities, the SS therapy aims to improve sensory processing and integration in individuals with MCS. These strategies are suitable for MCS patients, with invaliding sensory difficulties with minimal responses. Each strategy can be adapted to complement the rehabilitative program individualized education plan.

### 2.5. Statistical Analysis

Data analysis was conducted using IBM SPSS Statistics, Version 24 (IBM Corp., Armonk, NY, USA). The significance level for statistical tests was set at *p* < 0.05. For the analysis of neurophysiological and functional measures, we employed MANOVA models for repeated measures with a between-subject factor (group: experimental and control) and within-subject factors (phases: T0—pre-intervention baseline; T1—post-test). Moreover, for HR measures we utilized an ANOVA model for repeated measures with a between-subject factor (group: experimental and control) and a within-subject factor (section of interventions: from T1 to T10). In case of significant effects, the effect size of the test was reported, computed, and categorized according to data squared η2. Furthermore, to provide a comprehensive assessment, we applied paired t-tests within each group (CG T0-T1 and EG T0–T1) and independent t-tests between the two groups (CG vs. EG at T0 and T1) for clinical outcome measures. We assessed the assumption of normality using the Shapiro–Wilk test and examined the homogeneity of group variances using Levene’s test. The data analyzed had a normal distribution, and the test was not significant, so Student t-tests, using the Bonferroni correction, were used for post hoc testing of group differences in time and performance. Power analysis using Cohen’s d as the effect size parameter was applied.

## 3. Results

In total, 20 MCS patients, 5 females and 15 males with a mean age of 59.05 (±11.87), were enrolled and analyzed in this study. No side effects were reported at the end of the protocol. Notably, there was no requirement to involve nurses or physicians to address medical issues that might have arisen during the training. Comparing pre- and post-test scores, we observed significant changes across all neurophysiological outcomes (P300 and HR) and clinical outcomes (LCF and CSR-R) (Table 3), which were obtained as reported in the Materials and Methods Section (2.3. Outcome measures). However, the same statistical significance was not observed in the CG, which showed score improvements but without reaching statistical significance. Table 3 shows the means and standard deviations of P300 latency in experimental and control patients, along with the *p*-values of independent sample t-tests.

**Figure 2:**
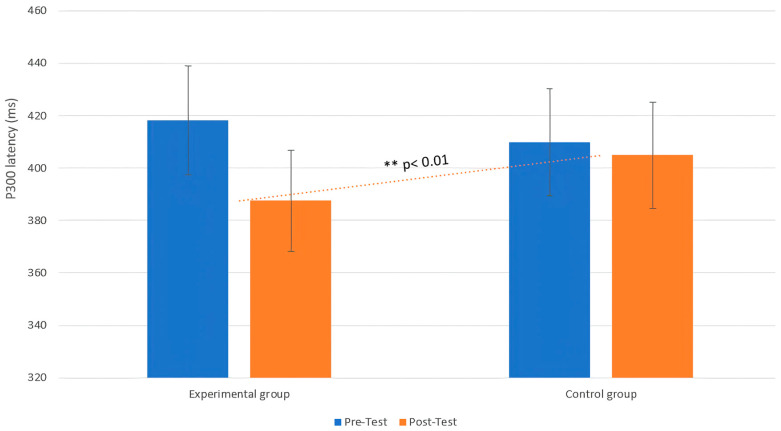
Changes in P300 latency between the pre-test (T0) and post-test (T1) phases in both the EG and CG. Legend: Comparison of P300 latency between the experimental group (EG) and the control group (CG) at both pre-test and post-test. The dotted trend line shows the significant difference in P300 latency between the groups at post-test, with a ** *p*-value < 0.01, indicating that the EG exhibited a distinct pattern in P300 latency compared to the CG only in the post-test.

A repeated MANOVA model, with group and phase as factors, was applied to assess the P300 latency. In particular, the MANOVA revealed a significant interaction effect between group and phase on P300 latency (F (1, 18) = 10.23, *p* < 0.001, η^2^ = 0.09), indicating that the intervention involving emotional tactile stimuli significantly influenced the P300 latency in the EG. Post hoc analysis showed that the experimental group exhibited a significant reduction in P300 latency from pre- to post-intervention (T0: M = 418.25 ms, SD = 63.43, 95% CI [380.24, 456.26]; T1: M = 387.57 ms, SD = 42.37, 95% CI [363.42, 411.72]; t = 6.81, *p* = 0.01, d = 0.79), while the CG showed no significant change (T0: M = 409.98 ms, SD = 58.11, 95% CI [373.12, 446.84]; T1: M = 404.99 ms, SD = 53.21, 95% CI [371.22, 438.76]; t = 1.06, *p* = 0.43, d = 0.69) (Figure 2).

**Figure 3:**
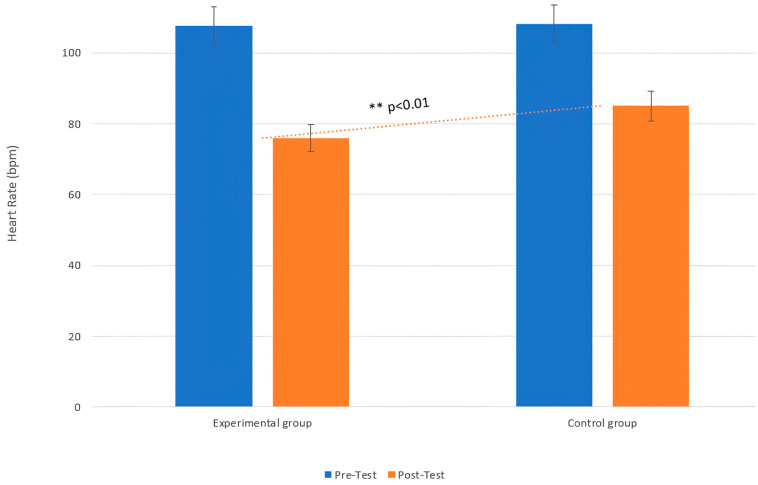
Changes in HR between the pre-test (T0) and post-test (T1) phases in both the EG and CG. Legend: Comparison of heart rate (HR) between the experimental group (EG) and the control group (CG) at both pre-test and post-test. The dotted trend line shows the significant difference in HR between the groups at post-test, with a ** *p*-value < 0.01, indicating that the EG exhibited a distinct pattern in heart rate compared to the CG only in the post-test.

Moreover, the MANOVA applied to HR revealed a significant interaction effect between group and phase (F (1, 18) = 18.27, *p* < 0.01, η^2^ = 0.09), indicating that the intervention involving gentle touch stimulation significantly influenced the HR in the EG. Post hoc analysis showed that the EG exhibited a significant reduction in HR from pre- to post-intervention (T0: M = 108.23 bpm, SD = 34.27, 95% CI [89.10, 127.36]; T1: M = 76.28 bpm, SD = 29.21, 95% CI [60.56, 92.00]; t = 11.89, *p* = 0.01, d = 0.85), while the CG showed no significant change (T0: M = 105.90 bpm, SD = 28.51, 95% CI [91.56, 120.24]; T1: M = 89.28 bpm, SD = 37.31, 95% CI [69.42, 109.14]; t = 12.05, *p* = 0.29, d = 0.77) (Figure 3).

### Clinical Parameters

**Figure 4:**
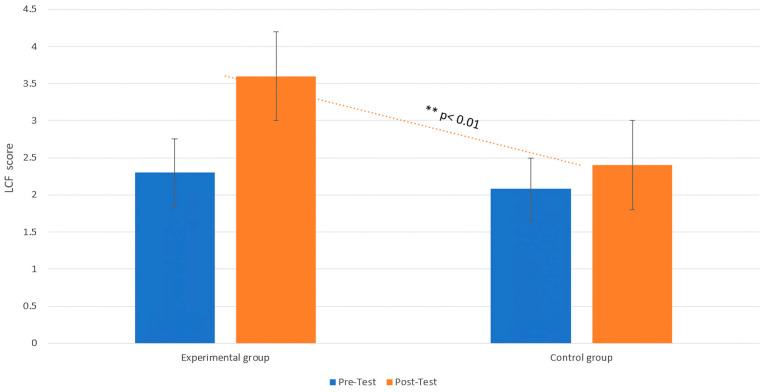
Changes in LCF between the pre-test (T0) and post-test (T1) phases in both the EG and CG. Comparison of the level of cognitive functioning (LCF) between the experimental group (EG) and the control group (CG) at both pre-test and post-test. The dotted trend line shows the significant difference in LCF scores between the groups at post-test, with a ** *p*-value < 0.01, indicating that the experimental group exhibited a distinct improvement in cognitive functioning compared to the control group only in the post-test.

Concerning LCF, the MANOVA revealed a significant interaction effect between group and phase (F (1, 18) = 5.99, *p* = 0.02, η^2^ = 0.09), indicating that the intervention involving gentle touch stimulation significantly influenced the LCF in the EG. Post hoc analysis showed that the EG exhibited a significant increment in LCF from pre- to post-interventions (T0: M = 2.30, SD = 0.67, 95% CI [1.93, 2.67]; T1: M = 3.60, SD = 1.03, 95% CI [2.98, 4.22]; t = 8.23, *p* = 0.01, d = 0.89), while the CG showed no significant changes (T0: M = 2.08, SD = 1.05, 95% CI [1.50, 2.66]; T1: M = 2.40, SD = 1.21, 95% CI [1.72, 3.08]; t = 1.05, *p* = 0.55, d = 0.85) (Figure 4).

**Figure 5:**
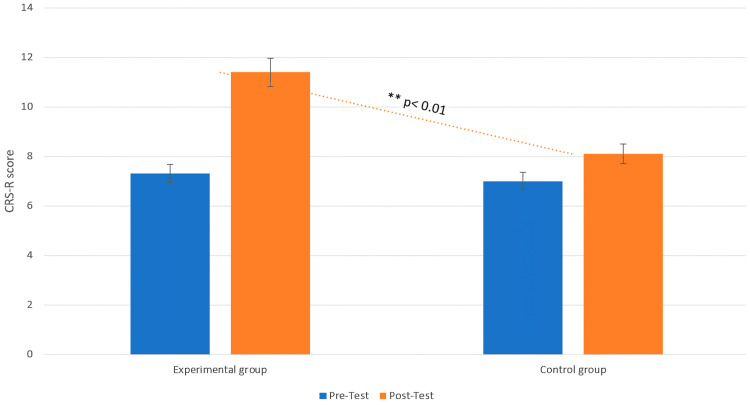
Changes in CRS-R between the pre-test (T0) and post-test (T1) phases in both the EG and CG. Legend: Comparison of the coma recovery scale—revised (CSR-R) between the experimental group (EG) and the control group (CG) at both pre-test and post-test. The dotted trend line shows the significant difference in CSR-R scores between the groups at post-test, with a ** *p*-value < 0.01, indicating that the EG exhibited a distinct recovery pattern compared to the CG only in the post-test.

Concerning CRS-R, the MANOVA revealed a significant interaction effect between group and phase (F (1, 18) = 6.45, *p* < 0.05, η^2^ = 0.11), indicating that the intervention involving tactile stimuli significantly influenced the CSR-R in the EG. Post hoc analysis showed that the EG exhibited a significant increment from pre- to post-intervention (T0: M = 7.30, SD = 2.54, 95% CI [5.95, 8.65]; T1: M = 11.30, SD = 4.80, 95% CI [8.71, 13.89]; t = 12.05, *p* = 0.01, d = 0.77), while the CG showed no significant change (T0: M = 7.00, SD = 2.58, 95% CI [5.59, 8.41]; T1: M = 8.10, SD = 2.91, 95% CI [6.52, 9.68]; t = 0.95, *p* = 0.39, d = 0.91) (Figure 5).

## 4. Discussion

In this study, we investigated the effects of gentle touch stimulation combined with advanced sensory stimulation delivered through the Neurowave device in people with MCS, compared to conventional SS. Our findings support the hypothesis that advanced sensory stimulation plus gentle touch has beneficial effects on various parameters (P300, HR and clinical outcomes). Although both groups (EG and CG) improved their neurophysiological and clinical outcomes, the EG gained better outcomes. These results could be explained by the fact that our combined protocol enriched the patient’s environment, promoting neural plasticity, and further improving patients’ recovery. The rationale for applying emotional and autobiographical stimuli has been confirmed by various authors [9,10]. This type of stimulation, which involves structured and meaningful stimuli, engages both input and output cognitive processes. Specifically, such stimuli are delivered through multiple sensory channels in an integrated and simultaneous manner, addressing cognitive processing in a dynamic and naturalistic way. In this way, alternating stimuli of different intensities helps maintain attention and interest, ensuring that the stimulations remain engaging and effective [9].

Among the senses, touch comprises sensory-discriminative and affective components. The affective component plays a crucial role in normal human development and everyday social interactions, influencing emotions like pleasantness and unpleasantness [25]. In our protocol, we used gentle touch stimulation, which can simply be referred to as “gentle touch”. This type of touch involves soft, tender physical contact, characterized by light pressure and delicate movements [5,7]. It has been shown that gentle touch, particularly on the hand, activates brain regions related to emotion and reward [7]. The recently discovered “CT fibers” have been found to project to the insular brain regions, identifying these afferents as part of an interoceptive system that provides information about the body’s affective and physiological state, enhancing body awareness [26,27,28]. Interoception, through its influence on sensory input, individual differences, learning and memory, brain injury recovery, and experience-dependent plasticity, may affect neuroplasticity [29], potentially contributing to our positive findings related to P300.

Moreover, gentle touch stimulation was combined with advanced sensory stimulation using an emotional approach, personalized to each patient. In a previous study [17], we applied this approach to a patient with severe traumatic brain injury, finding that it improved the patient’s behavioral responsiveness more effectively than traditional cognitive rehabilitation. In another study [10], we observed that a multisensory and emotional protocol using the Neurowave led to better outcomes in MCS patients than conventional training. However, since the Neurowave does not provide tactile responses, we incorporated touch to enhance the neurophysiological and clinical effects of the advanced multisensory stimulation with Neurowave. Emotional stimuli that capture attention, like those provided by the Neurowave in addition to gentle touch, are prioritized within the cognitive system, intensifying sensory integration. In this context, our study is the first to investigate the impact of the effects of advanced multi-sensory stimulation combined with gentle touch stimulation, compared to the conventional SS approach, on neurophysiological and functional outcomes in patients with MCS. Other studies investigated the role of the SS program, which mainly included auditory, visual, tactile, olfactory, and gustatory stimulation based on patients’ personal experiences and preferences, as reported in Table 4.

### 4.1. Neurophysiological Findings

Firstly, concerning neurophysiological responses, we observed a significant reduction in P300 latency in the EG following gentle touch stimulation. This finding suggests that advanced multisensory stimulation combined with gentle touch may enhance neural processing efficiency in response to cognitive tasks, as evidenced by the shortened P300 latency. In contrast, the CG did not show significant changes, highlighting the specificity of the intervention’s impact on neurophysiological responses. The brain’s response to the multi-sensory stimulation delivered through this innovative tool has been measured using the P300 wave. The P300, the third positive wave of ERP, is considered the most reliable cognition-related wave for assessing consciousness in DoC [33,34]. In this context, Li et al. [35] demonstrated that P300 can serve as a prognostic indicator, helping to identify patients with a higher likelihood of recovery. Neurophysiological evaluation via the P300 aids clinicians in both diagnosing and classifying DoC patients, as well as providing prognostic insights for their management. Thus, integrating Neurowave in clinical practice offers a customized rehabilitation strategy for patients with MCS, combining objective brain activity assessment with a personalized treatment approach. On the other hand, the use of tactile stimulation with emotional salience could be a feasible and valuable option for MCS patients’ treatment. From a neurophysiological point of view, emotional information is given priority in the cognitive system, with emotional stimuli gaining privileged access to attention and awareness [36]. Furthermore, emotion enhances sensory processing, amplifying the brain’s response to emotionally salient inputs [36]. Additionally, emotion influences the encoding of stimuli to be remembered, with arousal believed to enhance hippocampal-dependent consolidation processes [37]. To this aim, Ellingsen et al. [38], showed that the visual display of faces showing emotional expressions influenced participants’ perceptions of the pleasantness of simultaneous touch stimuli. Participants rated touch as most enjoyable when paired with an image of a smiling face and least enjoyable when paired with a frowning face [38]. Notably, this effect occurred even though participants knew the person in the photograph was not the one administering the touch. This finding suggests that affective, time-synchronized emotional visual cues—despite providing no specific information about the touch itself—can still shape the perceived pleasantness of the tactile experience. Regarding the interaction between touch and simultaneous non-touch signals, it has been suggested that touch can amplify the emotional impact of other sensory experiences [6]. Physical contact inherently signifies that someone or something is in direct contact, which often demands a quick response. This potential amplifying effect of touch on other sensory cues may help us quickly determine whether the person touching us is friendly or hostile—essential information when someone is nearby. In the context of DoCs, this could help in assessing residual cognitive function and responsiveness. For example, combining gentle touch with a familiar voice or a recognizable scent may elicit more discernible behavioral responses, which can guide clinicians in evaluating the patient’s level of consciousness and tailoring rehabilitation strategies.

Moreover, the use of gentle touch stimulation can also be used to prevent or reduce loneliness. In this sense, the COVID-19 era has made us realize how essential human contact is, not only among healthy individuals but even more so for those in ICUs, like MCS patients [39]. The absence of touch can lead to sensory deprivation, especially in this patient population. Gentle touch-based stimulation, an entirely non-invasive technique, can also help prevent isolation and the perception of abandonment. In this context, the caregiver’s presence could be incorporated into rehabilitation sessions to explore whether it influences neurophysiological and clinical outcomes. In this regard, Moattari et al. [16] investigated the role of SS delivered by patients’ family members. They found that patients who received SS from their family members showed a significant increase in their level of consciousness compared to those stimulated by nurses. This aspect could be explained by the fact that the reaction to a familiar face is given to increased salience due to “affective meaning” based on prior experiences, which causes the individual to recreate the experience, even if they are not fully experiencing it [39]. In this sense, the presence of patients’ family members in the context of SS programs could support the multidisciplinary rehabilitation team in promoting levels of consciousness and cognitive functioning [16,40]. Additionally, face-to-face interaction as well as physical contact could help relieve stress through social buffering. This aspect could explain the significant decrease in HR, which suggests a calming effect after the gentle touch stimulation. This observation aligns with our hypothesis that tactile interventions can modulate autonomic functions, potentially promoting relaxation and physiological stability in MCS patients. The sustained significance of HR reductions across multiple intervention sessions underscores the robustness of this effect. The rationale for this finding could be the fact that the gentle touch, by engaging CT fibers, directly influences the parasympathetic nervous system, promoting relaxation and reducing stress [3,26,27,41]. Previous studies demonstrated that the use of gentle touch in general (i.e., to glabrous skin and not just the C-tactile afferent innervated hairy skin) activates higher cortical areas, associated with the processing of emotion (e.g., perceived pleasantness), as found both in infants [7] and in adults [1]. Taken together, these data indicate that gentle touch processing is present in adults and evokes specific central and autonomic responses. However, other authors [42,43] have used different types of sensory stimuli, such as noise, demonstrating that these stimuli can enhance the coherence of neurophysiological signals at both the brain and spinal cord levels, either through multisensory stochastic resonance or gentle noise touch stimulation. Future research could investigate a wider range of sensory stimuli, such as noise, to further understand their role in enhancing neurophysiological signals. This could help identify the most effective types of stimuli for specific neural systems.

Furthermore, we hypothesized that the application of emotional gentle touch stimulation combined with Neurowave device would have a positive impact on HR of patients with MCS by lowering the rate, suggesting an autonomic calming effect. For example, Maseda et al. [44] found a significant reduction in HR after multisensory stimulation in patients with moderate and severe Alzheimer’s disease. Similarly, Machado et al. [45], found that a multisensory stimulation program in people with dementia reduced agitation and apathetic behavior. This aspect suggests that multisensory stimulation may promote positive emotions, increasing the interaction with the environment and redirecting to engaged behavior. In patients with DoC, multisensory stimulation seems to promote arousal and patient’s responsiveness. To this aim we found that HR was statistically reduced in the post-intervention compared to the pre-intervention, suggesting that our intervention provided a calming effect. In future studies, it may also be useful to record patterns of EEG cortical coherence, as they can serve as sensitive and early markers for predicting recovery in patients with DoC. For example, Schorr et al. [46] found that fronto-parietal and parietal coherence were associated with improvements in UWS patients, indicating a transition to MCS. DoC patients exhibiting higher theta and alpha coherence in parietal regions were identified as strong early indicators of recovery.

### 4.2. Clinical Findings

Regarding clinical outcomes (CRS-R and LCF), our results indicate significant improvements in awareness and cognitive functioning. Specifically, we observed enhancements in the CRS-R and LCF following experimental treatment. These improvements suggest that multisensory and emotional tactile stimulation may facilitate cognitive and functional recovery processes in MCS patients, possibly by enhancing neural responsiveness and promoting adaptive behaviors. It is conceivable that the use of an innovative tool, like the Neurowave, was able to furnish more intensive, repetitive, task-oriented, and emotional stimulation associated with touch-based stimulation, consequently boosting neuroplasticity and functional recovery. According to some authors, improvement in consciousness in patients with DoC not only depends on the type of stimulation (positive, negative vs. neutral stimuli) but also on the possibility of providing such stimulation intensively and repetitively, as the Neurowave can do. In addition, the presence of emotional content (e.g., the sight of a familiar face), as proposed in our experimental protocol, is known to promote oxytocin release, a hormone that controls key aspects of human behavior [38,47,48]. In addition, stimulation of cutaneous afferents from most parts of the body gives rise to oxytocin release and an oxytocin-linked effect spectrum [49]. Although we did not measure this hormone, future studies should investigate it to strengthen the clinical and neurophysiological findings related to the effects of gentle touch stimulation in DoC patients.

Gentle touch via activation of CT afferents is considered to be an important trigger of well-being in response to cutaneous stimulation, as such stimulation has been shown to activate areas within the anterior cingulate cortex, which is involved in positive emotions [5,50]. Moreover, the reaction to a familiar emotional content increases the salience of the stimuli, due to its “affective meaning” based on prior experiences, which causes the individual to recreate the experience, even if they are not fully experiencing it. A gentle touch could reinforce this aspect, adding more perception of physical awareness during the treatments.

### 4.3. Strengths, Limitations, and Future Perspectives

The strengths of our study are linked to the implementation of emotional and multisensory stimulation, which included gentle touch stimulation in patients with MCS. In general, the positive effects of gentle touch stimulation are documented in healthy subjects, infants or patients with chronic pain, but not in patients with MCS or in general DoC. According to our results, gentle touch stimulation could be a feasible, safe and complementary approach to use for patients with MCS. In addition, the use of neurophysiological parameters like P300 and HR in addition to functional outcomes (LCF, CRS-R) to investigate our hypothesis represents another strength.

It is noteworthy that the role of touch is often an overlooked aspect in clinical practice, especially in patients with acquired brain injury. There is an assumption that patients perceive the same tactile sensitivity as healthy individuals, without considering how the touch of a nurse, physician, or therapist might be perceived by the patient. Touch serves as a crucial means of communication for both the patient and the healthcare professional, particularly in cases where verbal communication is impaired, like in MCS patients. In this kind of patient population, the ability to communicate verbally may be limited or non-existent, and in these cases, touch can become one of the few remaining ways to establish a connection, convey comfort, or provide reassurance. However, touch is not simply a technical action, it carries emotional and psychological weight and can be interpreted in a variety of ways depending on the intent, pressure, and sensitivity of the healthcare professional and/or caregiver. Moreover, these patients are unable to move autonomously, often depending entirely on healthcare workers for basic activities of daily living such as feeding, hygiene, and repositioning. This dependence on others for physical contact raises the question of whether healthcare workers and caregivers are fully aware of the impact of their touch and how it is experienced by the patient. This is why it is essential to acknowledge that for patients who are unable to verbally express discomfort or communicate preferences, touch may be their only form of communication. A clinical touch—whether soft or firm, hurried or gentle—can convey a variety of messages, from empathy and respect to indifference or impatience. In this context, touch becomes a language of its own, and healthcare providers must be mindful of the significance of this non-verbal communication tool. A gentle touch has the potential to deliver feelings of comfort, safety, and trust, which are essential for the well-being of patients, particularly those with neurological injuries or impairments.

Thus, the inclusion of gentle touch in clinical settings has the potential to not only improve the patient’s emotional state but also to enhance the overall therapeutic environment. It can help create a more compassionate and human-centered approach to care. This use of touch could become an integral part of nursing and rehabilitation practice, transforming everyday interactions into opportunities for emotional connection and healing.

However, our research has some limitations that need to be acknowledged. First, the small sample size does not allow us to extend our promising results to the whole MCS population. Second, the use of a quasi-randomization procedure could have increased the bias in participant selection, especially in terms of participants comparison at baseline. However, our sample did not show statistically significant differences in the demographic parameters and clinical outcomes at the baseline. Moreover, another important limitation is the absence of long-term follow-up, which further prevents the generalizability of our results, since it becomes challenging to determine whether the effects of an intervention are sustained or only temporary. In addition, we focused solely on P300 latency, while the potential effects of the intervention on P300 amplitude were not assessed. Future research should explore P300 amplitude to provide a more comprehensive understanding of the impact of emotional multisensory stimulation on attentional resource allocation and neural activity. Nevertheless, it is essential to highlight that this study serves as an exploratory study, emphasizing the requirement for future clinical investigations with expanded sample sizes and refined neurophysiological, motor and cognitive outcome measures.

Future research could further explore the underlying mechanisms of tactile stimulation effects, optimize intervention protocols, and assess long-term outcomes to refine clinical practices. Additionally, it would be important to explore the effects of touch performed by the caregiver in comparison to that of a healthcare professional, as well as how the caregiver or the patient perceives gentle touch. Moreover, it could be interesting to investigate the role of gentle touch-based stimulation at the brain level with electroencephalogram, adding also the investigation of autonomic effects through heart rate variability, blood pressure, and oxytocin dosage.

## 5. Conclusions

The findings of this pilot study contribute to our understanding of the therapeutic potential of emotional multisensory stimulation, which also includes gentle touch stimulation, in MCS rehabilitation. By using both neurophysiological and functional measures, our results could support the integration of tactile interventions into comprehensive neurorehabilitation programs. In conclusion, our study suggests that gentle touch combined with advanced multi-sensory stimulation holds promise as a non-invasive and potentially effective intervention for improving neurophysiological functioning and functional outcomes in MCS patients. These findings pave the way for incorporating multimodal approaches, such as tactile stimulation, in enhancing rehabilitation outcomes, and maybe the quality of life, of individuals with severe neurological conditions.

## Figures and Tables

**Table 1:** Socio-demographic clinical description of the population’s study.

Patients	EG = 10	CG = 10	All Patients = 20	*p*-Value
Age	56.3 (12.87)	61.8 (10.73)	59.05 (11.87)	0.40
Gender				0.60
Female	2 (20%)	3 (30%)	5 (25%)	
Male	8 (80%)	7 (70%)	15 (75%)	
Education (years)				0.66
Elementary school	1 (10%)	3 (30%)	4 (20%)	
Middle school	3 (30%)	4 (40%)	7 (35%)	
High school	4 (40%)	3 (30%)	7 (35%)	
University	2 (20%)	0 (0.0)	2 (10%)	
Etiology				0.63
Traumatic	4 (40%)	3 (30%)	7 (35%)	
Vascular	6 (60%)	7 (70%)	13 (65%)	

Legend: EG (experimental group); CG (control group). Quantitative variables (e.g., age) are expressed as means (standard deviations), while categorical variables (e.g., gender, education, and etiology) are expressed as frequencies and percentages.

**Table 2:** Description of both experimental and conventional intervention.

Sensory Rehabilitation Program Focused on Tactile Stimulation for MCS		
MCS Sensory Rehabilitation	EG	CG
	Experimental intervention	Conventional intervention
METHODOLOGICAL APPROACH	NES (Emotional Neurowave Stimulation): advanced audio—video stimulation with autobiographical materials + Touch-Based stimulation, also named as “Gentle Touch” + Traditional Neurorehabilitation (*physiotherapy and speech therapy)*	CSS—(Conventional Sensory Stimulation) without the Neurowave Technology System, using neutral refractory audio-video materials, without biographical content + Neutral touch-based stimulation + Traditional Neurorehabilitation (*physiotherapy and speech therapy)*
Setting	Neurowave Room	Sensory Room
Duration of rehabilitation Sessions	60 min: 30 min of NES 30 min of Gentle—Touch	60 min: 30 min of CSS 30 min of Neutral touch-based
Duration of sensory rehabilitation program	3 times a week, for 24 consecutive weeks	3 times a week, for 24 consecutive weeks
Multidisciplinary team	Neurologist, Psychiatric Therapist, Physiotherapist, Nurse and Speech therapist	Neurologist, Psychiatric Therapist, Physiotherapist, Nurse and Speech Therapist
Outcome measures	Clinical and Vital Parameters, Psychometric and Neurophysiological indicators: HR (bpm)—P300 Latency (m/s)—LCF—CRS-R	Clinical and Vital Parameters, Psychometric and Neurophysiological indicators: HR—P300 Latency—LCF—CRS-R

Legend: HR (heart rate); LCF (level of cognitive functioning); CSR-R (coma recovery scale—revised).

**Table 3:** Mean, standard deviation, and t-tests of the parameters of the study in pre-test (T0) and in post-test (T1).

	Pre-Intervention (T0)	Post-Intervention (T1)			
	M (±SD)	M (±SD)	t	*p*	d
EG					
P300 Latency (ms)	418.25 (±63.43)	387.57 (±42.37)	6.81	0.01 **	0.79
HR (bpm)	108.23 (±34.27)	76.28 (±29.21)	11.89	0.01 **	0.85
CRS-R	7.30 (±2.54)	11.30 (±4.80)	12.05	0.01 **	0.77
LCF	2.30 (±0.67)	3.60 (±1.03)	8.23	0.01 **	0.89
					
CG					
P300 Latency (ms)	409.98 (±58.11)	404.99 (±53.21)	1.06	0.43	0.69
HR (bpm)	105.90 (±28.51)	89.28 (±37.31)	12.05	0.29	0.77
CRS-R	7.00 (±2.58)	8.10 (±2.91)	0.95	0.39	0.91
LCF	2.08 (± 1.05)	2.40 (±1.21)	1.05	0.55	0.85

Legend: HR (heart rate); CSR-R (coma recovery scale—revised); LCF (level of cognitive functioning). ** significant at *p* < 0.01.

**Table 4:** Summary of relevant evidence in the field of SS in patients with DoC. The table highlights the similarities and the differences between the literature and our study.

First Author and Year of Publication	Sample Size and Patients’ Etiology/Diagnosis	Sensory Stimulation Methods	Similarities/Differences
Cheng et al., 2018 [30]	Twenty-nine patients with VS and MCS	Multi-sensory stimulation program, included auditory, visual, tactile, olfactory, and gustatory stimuli. Each kind of stimuli was based on the preferences of the patient before injury.	Similarity: The authors employed a personalized multisensory program, based on patient’s personal experience. Differences: The authors used only a conventional type of SS (e.gt., pictures and music), and tactile stimulation was delivered with fingertips pressure on the patients’ upper body.
De Luca et al., 2023 [10]	Forty-two patients with MCS	Multi-sensory rehabilitation program with Neurowave or conventional.	Similarity: The authors used an advanced multi-sensory rehabilitation program with Neurowave to carry out a personalized SS program. Difference: The authors administered only audio-visual stimuli, without tactile stimulation.
Di Stefano et al., 2012 [31]	Twelve patients with DoC	SS program was based on biographically meaningful objects	Similarity: The authors administered an experimental multi-sensory stimulation program based on autobiographical and emotional stimuli, including gentle touch, auditory, visual and olfactory stimulation, as well as changes in the intensity and color of the environmental lighting. Difference: The authors used only objects of common use and not an advanced SS device, like Neurowave.
Moattari et al., 2016 [16]	Sixty patients post-traumatic brain injury	SS program was delivered by nurses or families.	Similarity: The authors administered an SS program, which included auditory, visual, and tactile stimuli. Differences: The authors included in their SS program olfactory stimulation. In addition, patients were stimulated by nurses or by their families.
Sargolzaei et al., 2017 [32]	Eighty patients with DoC	The SS program included auditory, visual, tactile, olfactory, and gustatory stimuli	Similarity: The authors administered a personalized SS program, based on the information provided by the patients’ family members. Difference: The authors used objects of common use to deliver the SS, and in addition they carried out gustatory and olfactory stimulation, which was not included in our protocol. Moreover, tactile stimulation was delivered through massage with olive oil, and not with gentle touch.

Legend: MCS (minimally conscious state); VS (vegetative state); DoC (disorder of consciousness); SS (sensory stimulation).

## Data Availability

The data presented in this study are available on request from the corresponding author. The data are not publicly available due to the privacy of research participants.
